# Supplementary material for: Functional and structural features of L2/3 pyramidal cells continuously covary with pial depth in mouse visual cortex
Source: Cereb Cortex. 2022 Aug 26;33(7):3715–33. doi: 10.1093/cercor/bhac303 (PMC10068292; doi:10.1093/cercor/bhac303)
Supplement: Supplementary_data_CC_second_bhac303 [file supplementary_data_cc_second_bhac303.docx]

**Supplementary data**

**Functional and structural features of L2/3 pyramidal cells continuously covary with pial depth in mouse visual cortex**

Simon Weiler*^1,2,4^, Drago Guggiana Nilo*^1,5^, Tobias Bonhoeffer^1,5^, Mark Hübener^1,5^, Tobias Rose^1,6^, Volker Scheuss**^1,3^


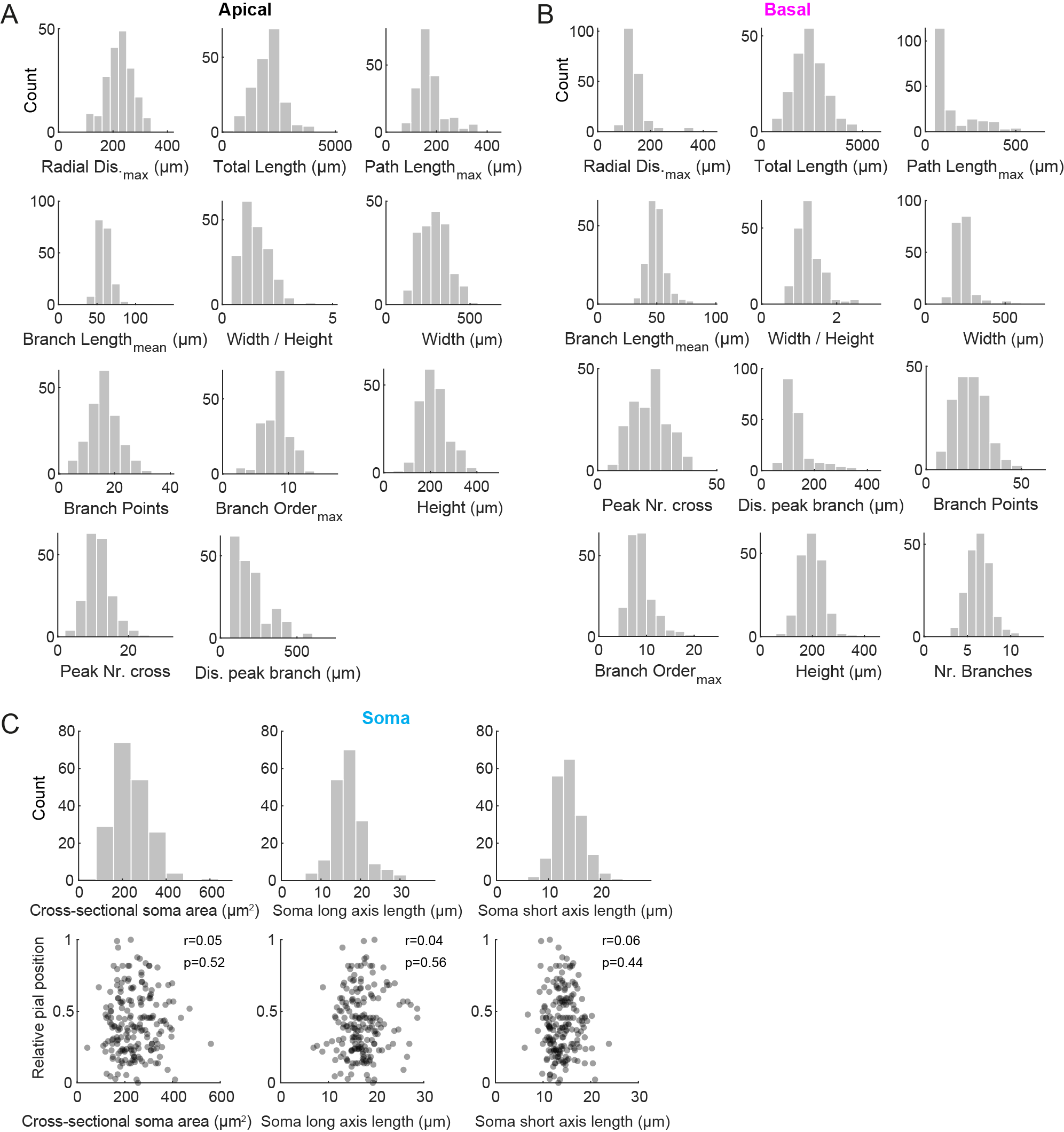


**Supplementary Figure 1** (A) Distribution of parameters used for morphological analysis of apical dendritic trees (n=189 cells, from 76 mice). (B) Same as (A) for basal dendritic trees. (C) Top: Distribution of soma parameters. Bottom: Relative soma position within L2/3 (0 - top, 1 - bottom of L2/3) plotted against soma cross-sectional area as well as vertical and horizontal soma diameter.


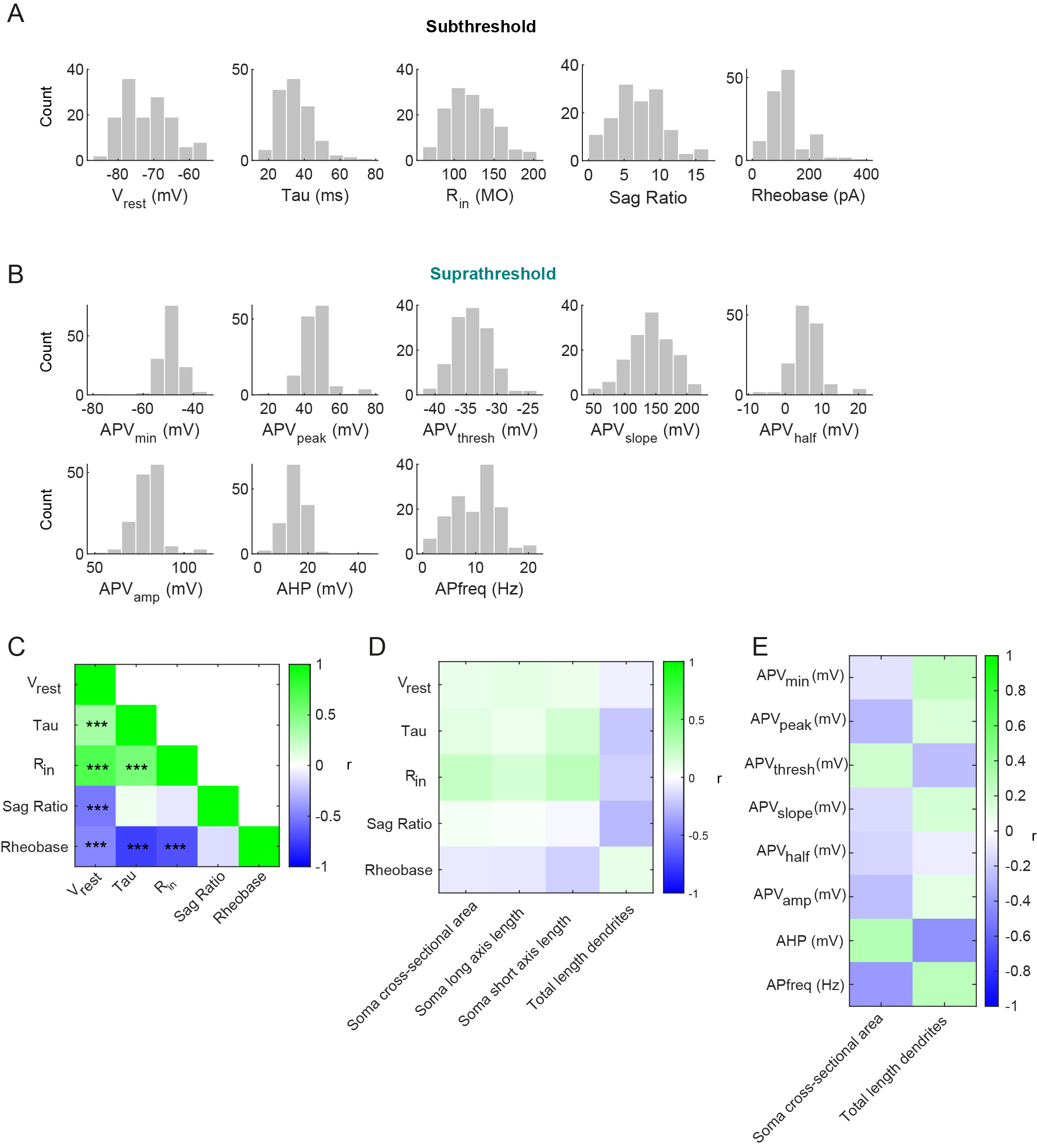


**Supplementary Figure 2** (A) Distribution of subthreshold (passive) properties (n=137, from 41 mice). (B) Same as A for suprathreshold (active) electrical properties. (C) Correlations between subthreshold properties (n=137, from 41 mice). Color indicates the Spearman’s correlation coefficient between the pair of parameters according to the color bar on the right Asterisks indicate significant correlations. (D) Correlations between morphological parameters for soma (cross-sectional area, soma short and long axis lengths) as well as total dendritic length and subthreshold intrinsic properties. (E) Correlations between morphological parameters for soma cross-sectional area as well as total dendritic length and suprathreshold intrinsic properties. V_rest_: Resting membrane potential; $\tau$_m_: Membrane time constant; R_IN_: Input resistance; Sag ratio: Sag in percentage; Rheobase: Minimal current necessary to evoke spike; APV_min_: Minimal membrane voltage during AHP; APV_peak_: Peak membrane voltage of spike; APV_thresh_: Threshold voltage at spike initiation; APV_slope_: The maximal slope of the spike; APV_half_: Membrane voltage at spike half; APV_amp_: Amplitude of the spike; AHP: Maximal amplitude of AHP; APfreq_max_: Maximal spike frequency.


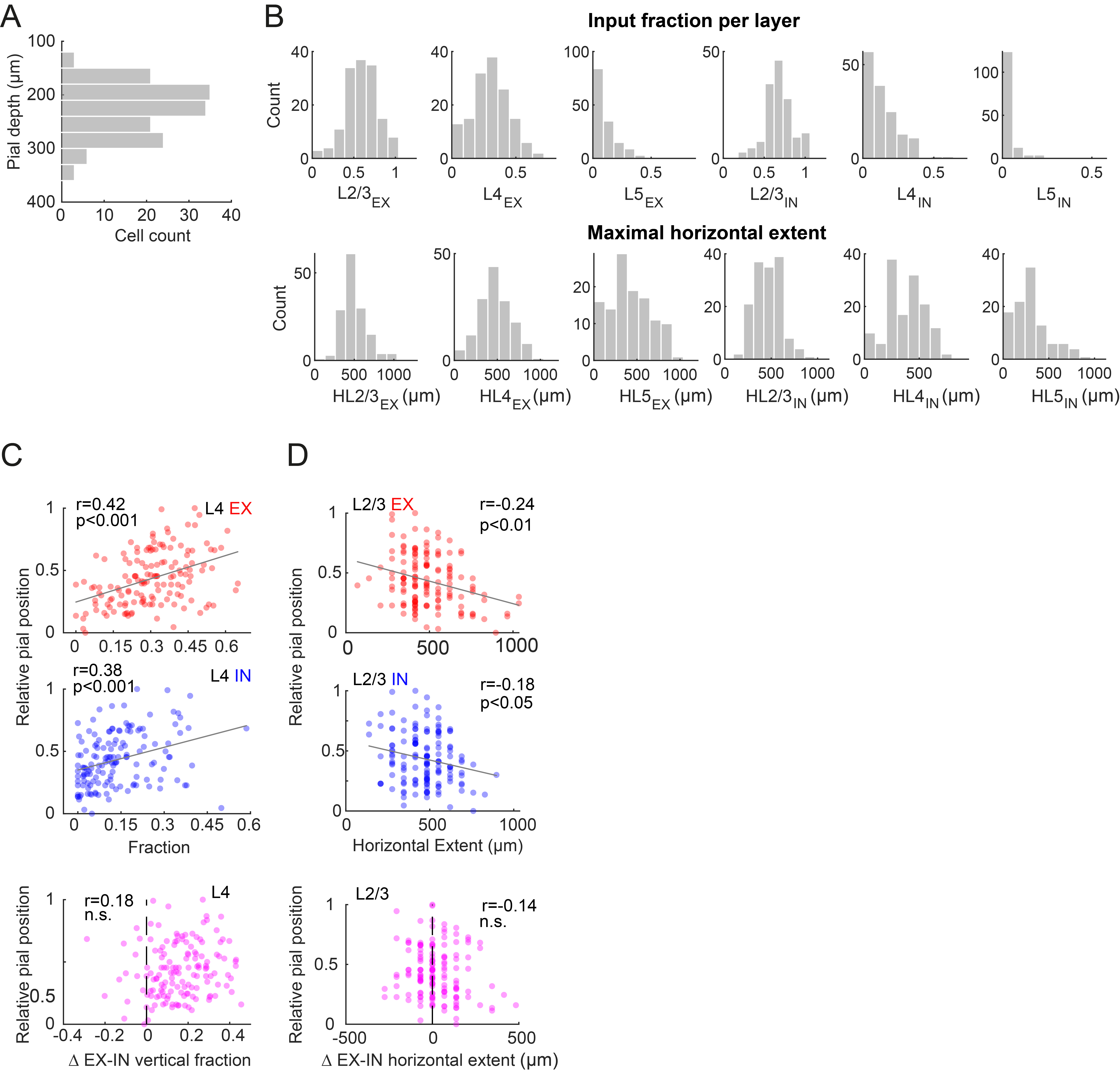


**Supplementary Figure 3** (A) Distribution of distances to the pial surface of functionally mapped neurons within L2/3 (n=147, from 56 mice). (B) Distribution of vertical and horizontal input map properties. (C) Relative pial depth plotted against excitatory (top) and inhibitory (middle) input fractions arising from L4 as well as difference of both (bottom) (n=147 cells, from 56 mice). Pearson correlation coefficient r indicated at top of each plot. Linear fit is indicated in grey. (D) Same as (C) for maximal horizontal extent of excitatory and inhibitory input from L2/3.


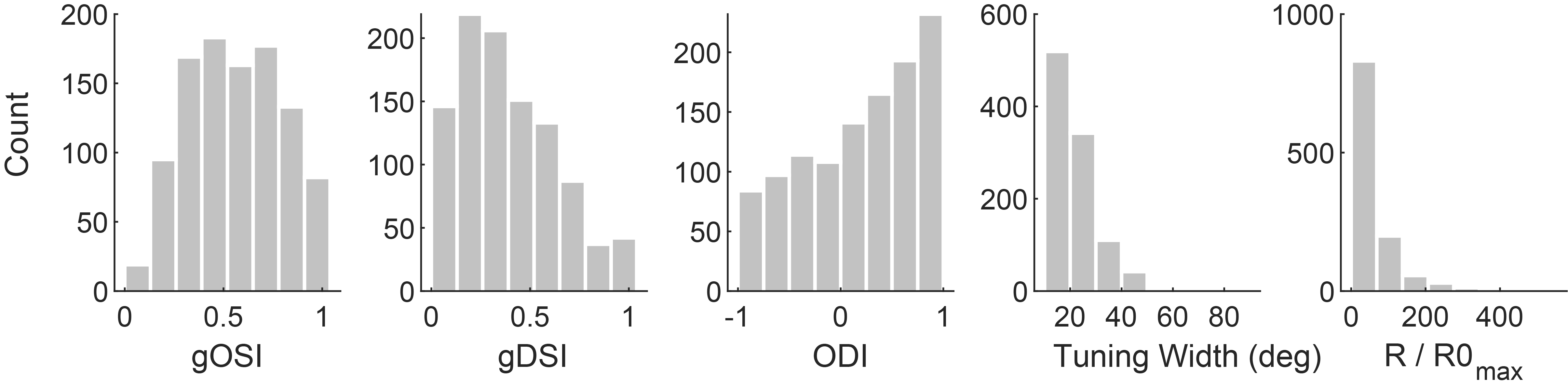


**Supplementary Figure 4** Distribution of visual response properties (gOSi= global orientation selectivity index, gDSI= global direction selectivity index, ODI= ocular dominance index, R/R0_max_= Maximal visually evoked response amplitude).


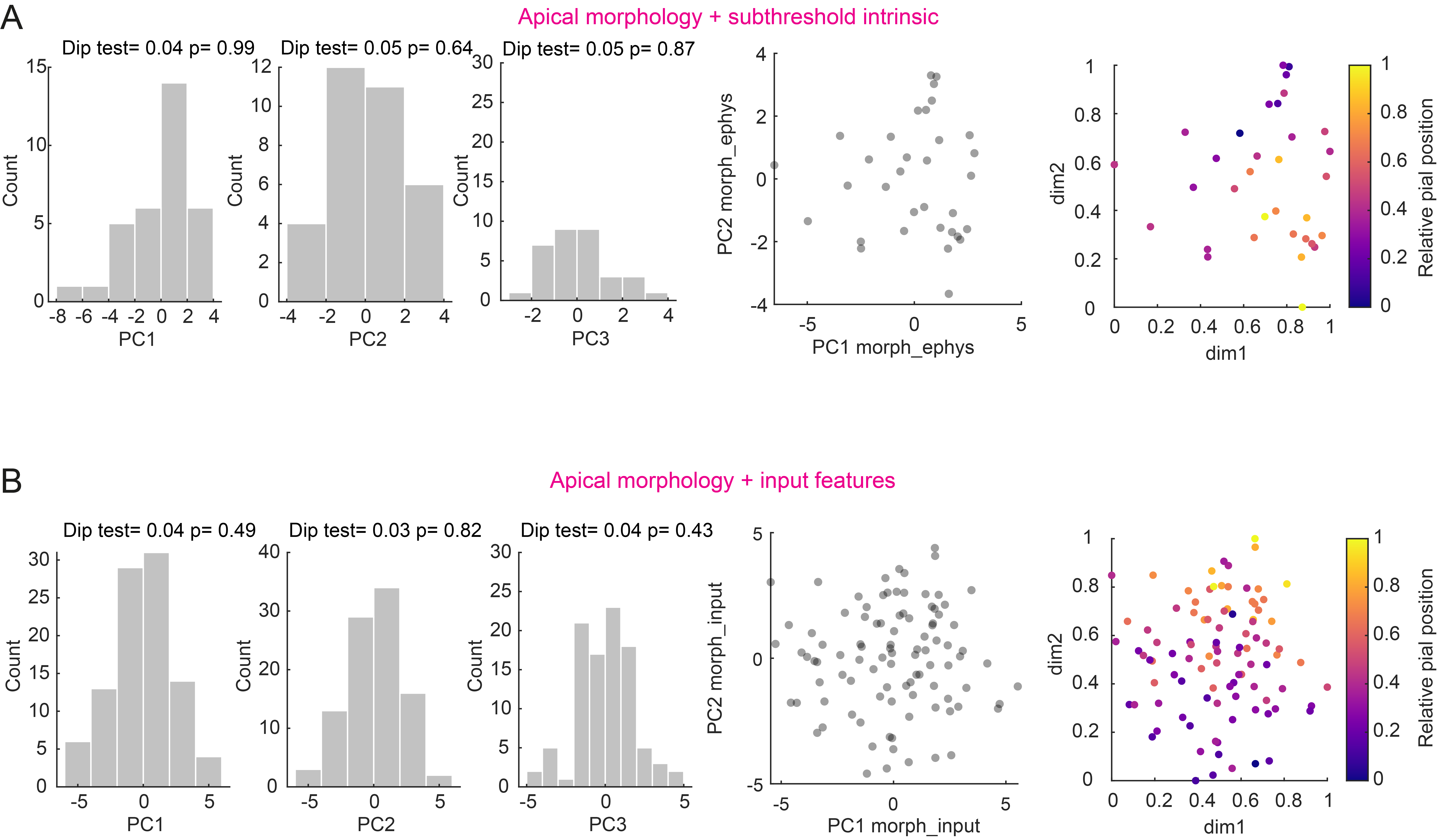


**Supplementary Figure 5** (A) Left, Distribution of principal component weights and Dip test results for multimodality for the first three principal components calculated for combined apical tree morphology and subthreshold properties. Middle, Principal component weights PC1 and PC2 of the combined data set plotted against each other (n=33 cells). Right, UMAP projections color-coded for relative pial position. The UMAP embedding was performed using the first three principal component weights of the combined data set. Dimension 1 (dm1) and 2 (dm2) are plotted. (B) Same as (A) for combined apical tree morphology and input connectivity properties. The following input map features were included in the PCA: Vertical excitatory and inhibitory fractions as well as horizontal excitatory and inhibitory extent for L2/3, L4 and L5 (see Supplementary Fig. 3).
